# The utility of albumin–bilirubin score in patients with intrahepatic cholestasis of pregnancy: a retrospective comparative study

**DOI:** 10.1590/1806-9282.20240860

**Published:** 2024-10-25

**Authors:** Osman Onur Ozkavak, Atakan Tanacan, Murat Haksever, Refaettin Sahin, Hakki Serbetci, Gulcan Okutucu, Eda Aldemir, Dilek Sahin

**Affiliations:** 1 Ankara Bilkent City Hospital, Department of Perinatology – Ankara, Turkey.; 2 University of Health Sciences, Ankara Bilkent City Hospital, Department of Obstetrics and Gynaecology – Ankara, Turkey.; 3 Ankara Bilkent City Hospital, Department of Obstetrics and Gynaecology – Ankara, Turkey.

**Keywords:** Albumin, Bilirubin, Intrahepatic cholestasis of pregnancy, High-risk pregnancy

## Abstract

**OBJECTIVE:**

The aim of this study was to examine the utility of the albumin–bilirubin score in cases of intrahepatic cholestasis of pregnancy.

**METHODS:**

A total of 413 patients (182 intrahepatic cholestasis of pregnancy, 50 suspected intrahepatic cholestasis of pregnancy, 181 healthy controls) enrolled in this study. Patients with typical pruritus and bile acid levels >10 μmol/L are defined as the intrahepatic cholestasis of pregnancy group. Patients with pruritus have the same pattern as intrahepatic cholestasis of pregnancy, but who are ultimately diagnosed with other dermatoses of pregnancy are defined as suspected intrahepatic cholestasis of pregnancy. Demographic data, laboratory parameters, and albumin–bilirubin scores were compared between three groups. Correlation analysis was performed on the albumin–bilirubin score and bile acid levels. Also, receiver operating curve analyses were performed to evaluate the predictive performance of the albumin–bilirubin score for intrahepatic cholestasis of pregnancy diagnosis.

**RESULTS:**

The albumin–bilirubin score of the intrahepatic cholestasis of pregnancy group was significantly higher than the other groups. A positive, weak correlation was found between the albumin–bilirubin score and bile acid levels in the intrahepatic cholestasis of pregnancy group. The receiver operating curve curve analyses showed albumin–bilirubin score has significant performance for the prediction of intrahepatic cholestasis of pregnancy in all subjects (area under the curve: 0.726, 95%CI 0.679–0.774, p<0.001) (sensitivity: 69%, specificity: 64%). The detection rate for albumin–bilirubin score was calculated as 67.3%. The positive predictive value was 3.95% (CI 2.9–5.3%), and the negative predictive value was 98.9% (CI 98.6–99.2%).

**CONCLUSION:**

This study indicated higher albumin–bilirubin score levels in the intrahepatic cholestasis of pregnancy group and a positive relationship between serum bile acid levels and albumin–bilirubin score. Therefore, albumin–bilirubin score could be a cost-effective liver function test for pregnant women with intrahepatic cholestasis of pregnancy.

## INTRODUCTION

Intrahepatic cholestasis of pregnancy (ICP) is the most common pregnancy-specific hepatic disease^1^. The major symptom is pruritus, which can affect the whole body, but palms and soles are especially affected^2^. The severity of pruritus can be moderate to severe, and it usually worsens at night^2^. Other symptoms may be right upper quadrant pain, nausea, and sleeping disorders. Typically, the disease has no pathognomonic findings.

Elevated serum total bile acid level is the mainstream of the diagnosis^3^. Alanine and aspartate aminotransferase levels may also increase^4^. Moreover, alkaline phosphatase and gamma-glutamyl transferase levels could increase too, but these findings are not specific to ICP.

In ICP patients, elevated serum bile acids transport from the placenta and accumulate in the amniotic fluid and fetal tissues^5^. This accumulation could lead to complications like meconium-stained amniotic fluid, sudden fetal demise, and neonatal respiratory distress syndrome^4^. Iatrogenic or spontaneous preterm birth rates are also increased in ICP patients^6^.

The albumin–bilirubin (ALBI) score is a scoring system, which was first developed for the assessment of liver function and prediction of prognosis in hepatocellular carcinoma^7^. The scoring system uses only the serum albumin and total bilirubin levels, which make this system useful and cost-effective. The prognostic value of the ALBI score in hepatocellular carcinoma patients who have undergone hepatic resection, and systemic or local ablative therapies has been shown in several studies^7,8,9^. Also, the prognostic value of the ALBI score in other hepatic and non-hepatic diseases is a new research area, and reports from the studies show that the ALBI score could be useful in the prediction of prognosis^10^.

Poor obstetric outcomes and higher rates of obstetric complications are found to be associated with ICP. If ICP is managed appropriately, favorable perinatal outcomes can be achieved. However, the diagnosis of ICP may be challenging, and evaluation of serum bile acid levels may not be possible in low-income settings. Thus, using a more practical index may help physicians in their clinical management protocols^4^.

Our study aimed to evaluate the value of the ALBI score as an ancillary liver function test and its ability to predict bile acid levels, and therefore the severity of the disease.

## METHODS

The study was designed as a retrospective case–control study. Patients who were admitted to the perinatology clinic of a tertiary research hospital between 12.10.2020 and 15.05.2023 were included in the study. Data were collected from the electronic database of the hospital. The institutional ethical committee approved the study under approval number E2-23-4758.

Patients with known cardiovascular, autoimmune, or endocrine disease, liver–gallbladder disease, acute or chronic renal disease, known malignant disease, malnutrition, multiple pregnancies, assisted reproductive technology pregnancies, hypertensive disorders of pregnancy, hemolysis, elevated liver enzymes, and low platelet (HELLP) syndrome, smokers, alcohol users, and patients who use progestins were excluded from the study. All participants were between the ages of 18 and 45.

Participants were divided into three groups. The first group was the ICP group, and patients with symptoms and serum bile acid levels >10 μmol/L were included in this group^11^. The second group was the suspected ICP group. Patients who had symptoms similar to ICP (pruritus and worsening of symptoms at night) but did not have elevated serum bile acid or other liver function test levels and had a final diagnosis other than ICP were enrolled in this group. A randomly selected frequency-matched low-risk pregnant women were used to form the control group.

The demographic information and laboratory results were gathered from the hospital’s patient record system. The gestational age of the patients was determined through the measurement of the crown-rump length, which is typically performed between the 11th and 14th gestational weeks. Clinical and demographic features (maternal age, gravidity, parity, gestational age at birth, birth weights, APGAR scores), laboratory findings at the time of the first application [hemoglobin level, white blood cell count (WBC), platelet count (PLT), albumin, total bilirubin], and ALBI scores were compared between the three groups. ALBI scores were calculated by using the (log10 bilirubin x 0.66)+(albumin x -0.085) formula^7^. All blood samples were collected at 30th–32th weeks of gestation for all participants.

Also, correlation analysis was made to explore the correlation between ALBI score and serum bile acid levels. The performance of the ALBI score for prediction of ICP in all patients was evaluated. The predictive value of the ALBI score for ICP in the symptomatic group was also assessed.

Statistical analyses were performed by using Statistical Package for the Social Sciences (SPSS.22, IBM SPSS Statistics for Windows, Version 22.0. Armonk, NY: IBM Corp.). We used the Kolmogorov-Smirnov test for the assessment of normality. Because the data were not normally disturbed, we used non-parametric tests and used median and minimum–maximum values for descriptions. Kruskal-Wallis test was performed to compare data between the groups. Spearman correlation analysis was used to evaluate the correlation between ALBI score and serum bile acid levels. Receiver operating curve (ROC) analysis was used for the estimation of the predictive value of the ALBI score for ICP. We made another ROC analysis for the evaluation of the predictive performance of the ALBI score in patients with symptoms (ICP and suspected ICP groups).

P-values less than 0.05 are accepted as statistically significant.

## RESULTS

**Figure 1:**
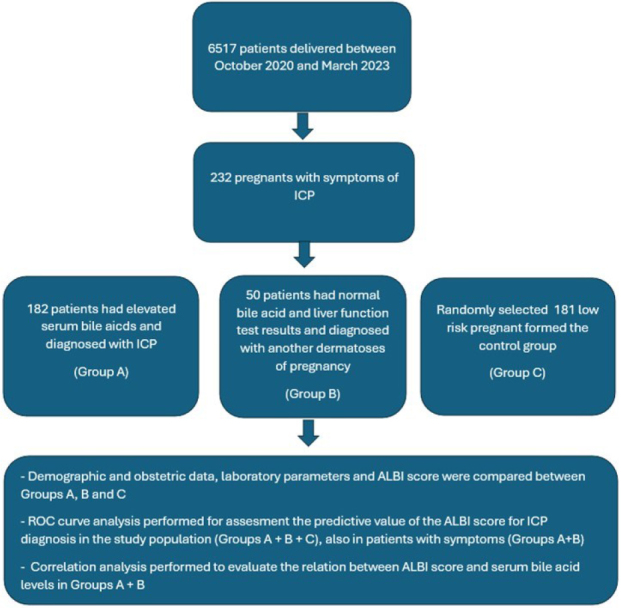
The flowchart of the study design.

A total of 413 patients were enrolled in the study, out of which 182 patients were grouped as ICP, 50 patients were grouped as suspected ICP, and 181 patients were grouped as the control group. Figure 1 shows the flowchart of the study design.

Birth weight in the ICP group was significantly lower than in the suspected ICP and control groups (p=0.02 and p<0.001, respectively). The 1st min. APGAR score in the ICP group was significantly lower than in the control group (p=0.015). The albumin level in the ICP group was significantly lower than in the suspected ICP and control groups (p=0.001 and p<0.001, respectively). The total bilirubin level of the control group was significantly lower than the ICP and suspected ICP groups (p<0.001 and p=0.011, respectively). Total bilirubin levels of the ICP and suspected ICP groups were not significantly different.

The ALBI score of the ICP group was significantly higher than both other groups (p=0.009 and p<0.001, respectively). There was no significant difference in ALBI score between suspected ICP and control groups. Comparison of demographic features and laboratory results between ICP, suspected ICP, and control groups is shown in Table 1.

**Table 1:** Comparison of demographic features and laboratory results between intrahepatic cholestasis of pregnancy, suspected intrahepatic cholestasis of pregnancy, and control groups.

	ICP (Group A) (n=182) Median (min–max)	Suspected ICP (Group B) (n=50) Median (min–max)	Control (Group C) (n=181) Median (min–max)	p-value		
				A vs B	A vs C	B vs C
Age	29 (18–45)	31 (21–44)	29 (19–45)	0.42		
Gravidity	2 (1–10)	1 (1–5)	2 (1–8)	0.87		
Parity	1 (0–4)	0 (0–3)	1 (0–6)	0.24		
Gestational age at birth (weeks)	37 (30–40)	38 (34–40)	38 (31–41)	0.06		
Birth weight (grams)	2,940 (610–4,105)	3,125 (2,120–4,500)	3,240 (1,780–4,555)	**0.02**	**<0.001**	0.42
1st. min APGAR score	7 (1–9)	8 (4–8)	7 (4–8)	**0.016**	0.174	0.135
5th. min APGAR score	9 (4–10)	9 (6–10)	9 (5–10)	0.17		
Hemoglobin (g/dL)	11.6 (8.1–14.5)	11.8 (9.3–14.8)	11.8 (7.2–15.9)	0.29		
White blood cell count (x10^9^/L)	9.03 (3.49–21.53)	9.29 (5.25–20.62)	10.02 (5.92–22.51)	0.42	**0.015**	0.3
Platelets (x10^9^/L)	246 (63–579)	244 (120–649)	257 (13–682)	0.77		
Albumin (g/L)	36.5 (26–44)	38 (9–50)	38 (31–43)	**0.001**	**<0.001**	0.61
Total bilirubin (mg/dL)	0.6 (0.1–6.2)	0.5 (0.2–8)	0.4 (0.1–1.7)	0.14	**<0.001**	**0.011**
ALBI score	-2.42 (-1.3 to -3.27)	-2.55 (-1.39 to -3.36)	-2.68 (-1.77 to -3.05)	**0.009**	**<0.001**	0.08

ICP: intrahepatic cholestasis of pregnancy; ALBI: albumin–bilirubin; g/L: grams per liter; mg/dL: milligrams per deciliter; p<0.05 accepted as statistically significant. The values that are statistically significant are indicated in bold.

Correlation analysis between ALBI score and fasting bile acid levels in the ICP group demonstrated a positive relationship between these parameters (p=0.003). The r value was 0.186.

**Figure 2:**
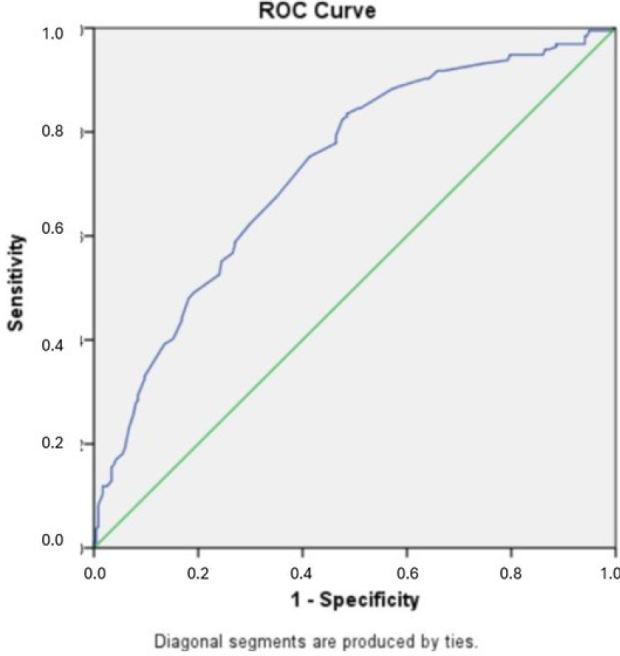
Receiver operator characteristics analysis for the prediction of intrahepatic cholestasis of pregnancy in all patients.

**Figure 3:**
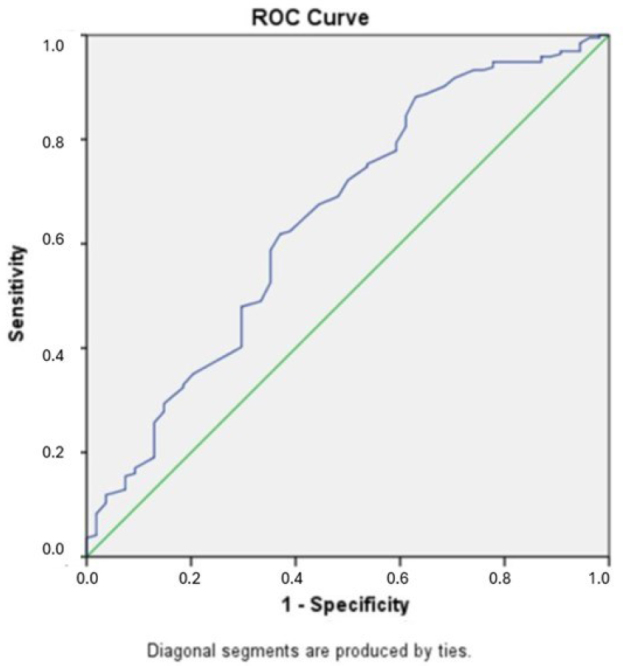
Receiver operator characteristics analysis for the prediction of intrahepatic cholestasis of pregnancy in suspected patients with symptoms.

In the ROC analysis for the predictive value of the ALBI score for ICP in all subjects, the area under the curve (AUC) was calculated as 0.726 (95%CI 0.679–0.774, p<0.001) and cutoff for the ALBI score with the best sensitivity and specificity was -2.53 (sensitivity: 69%, specificity: 64%). The detection rate for ALBI score in the whole study population was calculated as 67.3%. The positive predictive value was 3.95% (CI 2.9–5.3%) and the negative predictive value was 98.9% (CI 98.6–99.2%). The ROC analysis results for the predictive performance of ALBI score for patients with symptoms suspected of ICP, the AUC was 0.649 (95%CI 0.563–0.736, p=0.001), and the cut-off value was determined as -2.5 (sensitivity: 62%, specificity: 61%). The positive predictive value was 3.14% (CI 2.38–4.19%) and the negative predictive value was 98.7% (CI 98.3–99%). Also, the detection rate was 61.6% in the symptomatic group. Figures 2 and 3 show the ROC analysis results.

## DISCUSSION

The principal findings of the present study indicate that the novel marker ALBI score may prove advantageous in the diagnosis and assessment of the severity of ICP. Significantly higher ALBI score values were observed in ICP cases with a high negative predictive value, suggesting that this difference may be useful in differential diagnosis.

ICP affects 0.5–2% of pregnancies worldwide and is related to poor perinatal outcomes like stillbirth, meconium-stained amnion fluid, and preterm birth^12^. Patients usually present with pruritus, and usually, there are no other symptoms or physical examination findings. The best diagnostic clue is elevated serum bile acid levels defined as >10 μmol/L^13^. Other liver function enzymes could change in this situation, but they are not specific to ICP^4^.

In patients with ICP, it is recommended to plan the timing of delivery earlier than the standard schedule, taking into consideration the levels of serum bile acids^14^. At the aforementioned clinical facility, this practice is observed with the primary objective of reducing perinatal mortality. Our findings revealed that the infants in the ICP group exhibited a lower mean birth weight compared to the other two groups. It is hypothesized that this phenomenon is a consequence of ICP patients undergoing labor at an earlier gestational age than that observed in healthy pregnancies.

Albumin is a crucial plasma protein synthesized by the liver^15^. In the evaluation of liver functions, particularly in the context of chronic liver diseases, hypoalbuminemia serves as a pivotal parameter. Serum albumin level is subject to influence by various factors, including nutritional factors, catabolic processes like malignancies, and gastrointestinal or urinary losses^15^. Serum albumin levels tend to decrease during the pregnancy period, especially in the first trimester, primarily due to physiological hemodilution^16^.

In the current study, there was no significant difference in terms of gestational ages among the groups, and individuals with systemic diseases such as chronic liver diseases or malignancies, and patients with hypertensive disorders of pregnancy or known renal diseases were excluded from the study. Nevertheless, the albumin level of the ICP group was significantly lower than in the other two groups. This outcome may suggest that in ICP cases, albumin synthesis may be impaired due to the accumulation of bile acids in hepatocytes.

Serum total bilirubin levels do not change remarkably during the pregnancy period, thus, elevated bilirubin levels should be evaluated^17^. In ICP patients, 10% of cases have elevated serum bilirubin concentrations^17^. Similar to the literature, ICP cases had higher bilirubin levels compared to the controls in the present study. This finding may reveal that mild liver function alterations may occur in ICP.

The ALBI score was first described as a liver function test and prognostic marker for hepatocellular carcinoma patients^7^. In the subsequent phase, the applicability of this scoring system in liver diseases beyond HCC was investigated. Various studies have demonstrated the utility of the ALBI score as a prognostic marker in chronic viral or autoimmune hepatitis, as well as primary biliary cholangitis^18,19,20^. Another study showed that the ALBI score could help to evaluate fluid overload, liver dysfunction, and prognosis in acute heart failure patients^21^. An important advantage of the ALBI scoring system is its applicability even in the early stages of liver damage^10^. The present study demonstrated that the ALBI score in ICP cases was significantly higher than in both the other two groups.

Serum bile acid levels could be determined by different laboratory techniques. Mass spectrometry and liquid chromatography are commonly used tests, which are usually conducted by specialized laboratories, and the results are typically available within 4–14 days, depending on the technique^22^. The present study has revealed a positive correlation between the ALBI score and serum bile acid levels. In light of these findings, it seems reasonable to suggest that in healthcare facilities where suitable laboratory conditions for serum bile acid testing are not available, the ALBI score could serve as an additional liver function test in patients with ICP and could be beneficial for preventing delays in diagnosis and treatment. Symptomatic cases with higher ALBI scores may be referred to advanced health-care facilities earlier. Also, there is no consensus about the diagnostic criteria for ICP between guidelines, cut-off values for bile acid levels are different, and increased gamma-glutamyl transferase or aminotransferase levels are diagnostic in some, but non diagnostic in others^14,22,23^. Therefore, the ALBI score may have a supportive role in the diagnosis of ICP and may provide a useful tool for clinicians in the decision-making process.

To sum up, ALBI seems to be beneficial in the management of ICP in addition to traditional laboratory tests and clinical findings. However, future research including a higher number of participants is necessary to confirm the utility of the ALBI score in ICP cases.

The limitations of this study include its retrospective design and the fact that the data were obtained from a single center. The study’s strength lies in the number of patients included and the rarity of similar studies conducted in pregnant individuals.

## CONCLUSION

The ALBI score may be used as an ancillary test in the management of ICP cases. Physicians can easily use this novel index in their routine clinical practice. On the contrary, more data is needed to expand the application of ALBI in obstetric care.
